# Supplementary material for: Modeling of waning immunity after SARS-CoV-2 vaccination and influencing factors
Source: Nat Commun. 2022 Mar 28;13:1614. doi: 10.1038/s41467-022-29225-4 (PMC8960902; doi:10.1038/s41467-022-29225-4)
Supplement: Supplementary file 1 — Supplementary Information [file 41467_2022_29225_MOESM1_ESM.pdf]

Modeling of waning immunity after SARS-CoV-2 vaccination and influencing factors
Supplementary Information

Table of contents

Supplementary Methods 3
1. Data exclusion and modification before statistical analysis 3
2. Antibody response modeling 3
2.1. IgG antibody levels: Zero-inflated Gaussian mixed model 3
2.2. IgG antibody levels: Linear mixed model 4
2.3. IgM and IgA antibody responses: Binomial generalized mixed model 4
2.4. Neutralizing capacity index: Binomial generalized mixed model 4
2.5. IgG antibody levels associated to BMI: Zero-inflated Gaussian mixed model 5
2.6. IgG antibody levels associated to the interval of days between BNT162b2 vaccine doses: Zero-inflated Gaussian mixed model 5
2.7. IgA antibody responses associated to the interval of days between BNT162b2 vaccine doses: Binomial generalized mixed model 6
2.8. IgG antibody levels associated to IFN-γ levels: Zero-inflated Gaussian mixed model 6

Supplementary Figures 7
Supplementary Figure 1. Schematic illustration of sample collection timeline and the administration of the vaccine doses. 7
Supplementary Figure 2. Correlation of the BMI and circulating IgG levels against RBD after the first dose of the COVID-19 vaccination. 8
Supplementary Figure 3. Dynamics and projection with prediction intervals of circulating IgG levels against RBD after the first dose of the COVID-19 vaccination using a non-linear model. 9
Supplementary Figure 4. Observed levels and positive responses of circulating IgG, IgM, and IgA against RBD after the first dose of the COVID-19 vaccination. 10
Supplementary Figure 5. Observed and predicted probability of positive IgM responses against RBD after the first dose of the COVID-19 vaccination. 11
Supplementary Figure 6. Effect of the time of administration of the second dose of the BNT162b2 vaccine in the dynamic of circulating IgG levels against RBD. 12
Supplementary Figure 7. Effect of the time of administration of the second dose of the BNT162b2 vaccine in positive IgA responses against RBD. 13
Supplementary Figure 8. Correlation between released IFN-γ from activated T-cells upon recognition of peptides derived from the S1 subunit of S protein and IgG levels. 14
Supplementary Figure 9. Distribution of IFN-γ levels from activated T-cells upon recognition of peptides derived from the S1 subunit of S protein. 15

Supplementary Tables 16
Supplementary Table 1. RT-PCR responses in natural infection primed individuals 16

Supplementary Table 2. Multiple linear regression analysis for IFN-γ levels associations with sex, previous infection, age, IgG levels, and IgA responses in vaccinated individuals 17

Supplementary References 18

Supplementary Methods

1. Data exclusion and modification before statistical analysis

For those individuals who had a natural infection and the baseline sample was collected more than 14 days before the administration of the first vaccine dose, the baseline time point was excluded from the analysis. Individuals with two or fewer visits were excluded from the analysis. A total of 8 individuals vaccinated with a first dose of the ChAdOx1 vaccine and received a second dose of the Spikevax vaccine (Moderna) were excluded due to the low sample number (n=8). For the same reason, an individual fully vaccinated with the ChAdOx1 vaccine was excluded from the analysis. The days from baseline to the vaccine were reset to 0 if the individual did not have a natural infection prior to vaccination and the baseline sample was collected before the vaccine date. Other R packages used in this study were dplyr, tidyr, lubridate, table1, glmmTMB, lmerTest, splines, car, lme4, ggpubr, effects, wesanderson, ggthemes, ggnewscales, gridExtra, cowplot and forcats.

2. Antibody response modeling

The different mixed models used to study the different antibody levels and responses over time were performed using the glmmTMB R package [1]. The model checking and diagnostics of the residuals of the different mixed models were performed using the DHARMa R package (https://CRAN.R-project.org/package=DHARMa).

2.1. IgG antibody levels: Zero-inflated Gaussian mixed model

We utilized zero-inflated Gaussian mixed models due to the high proportion of samples with no detectable levels of IgG at baseline. Without this correction, it would not be possible to assume that the IgG levels data were normally distributed. This is a two-part model, one part is the conditional model that fits a Gaussian mixed model for the non-zero observations (detectable levels of IgG antibody), and a zero-inflated model that fits the binary model for the probability of observing a zero or not (detectable vs non-detectable IgG antibody levels).

We fitted the log10-transformed IgG levels in the zero-inflated Gaussian mixed model with subject level random effects for both intercept and slope across days from vaccination. The days from vaccination were represented with four natural cubic splines (NCS) to allow the modeling of non-linear trends. The knots of the four NCS were defined based on the 25%, 50%, and 75% quantiles of the days from vaccination. We

fitted the model that included the four NCS, N protein seropositivity, age groups, vaccine administered, and sex. In addition, we included a two-way interaction between NCS and N protein seropositivity, NCS and age groups, NCS and vaccine administered, and between N protein seropositivity and age groups to evaluate the IgG dynamics over time. We did not include an interaction between NCS and sex due to a lack of significance. For the zero-inflated model, we included days from vaccination, N protein seropositivity, and sex as the independent variables.

2.2. IgG antibody levels: Linear mixed model

We utilized linear mixed models to model the IgG levels from day 7 after the second vaccine as an input. We fitted the log10-transformed IgG levels in the linear mixed model with subject level random effects for both intercept and slope across days from the second vaccine. We fitted the model that included the days from the second vaccine, N protein seropositivity, age groups, vaccine administered, and sex. In addition, we included a two-way interaction between days from the second vaccine and N protein seropositivity, days from second vaccine and age groups, days from second vaccine and vaccine administered, and between N protein seropositivity and age groups to evaluate the IgG dynamics over time.

2.3. IgM and IgA antibody responses: Binomial generalized mixed model

We utilized binomial generalized mixed models to model the IgM and IgA responses over time after vaccination. We fitted binary responses of positive IgM and IgA responses with subject level random effects for intercept. The days from vaccination were represented with four NCS to allow the modeling of non-linear trends. The knots of the four NCS were defined based on the 25%, 50%, and 75% quantiles of the days from vaccination. We fitted the model that included the four NCS, N protein seropositivity, age groups, vaccine administered, and sex. In addition, we included a two-way interaction between NCS and N protein seropositivity, NCS and age groups, NCS and vaccine administered, and between N protein seropositivity and age groups to evaluate the IgM and IgA responses over time.

2.4. Neutralizing capacity index: Binomial generalized mixed model

We utilized binomial generalized mixed models to model the neutralizing capacity index over time after vaccination. We fitted binary responses of positive neutralizing capacity index with subject level random effects for intercept. We fitted the model that included the days from vaccination, N protein seropositivity, age groups, vaccine administered, and sex. In addition, we included a two-way interaction between days and

N protein seropositivity, days and age groups, days and vaccine administered, and between N protein seropositivity and age groups to evaluate the neutralizing capacity index over time.

2.5. IgG antibody levels associated with BMI: Zero-inflated Gaussian mixed model

We utilized zero-inflated Gaussian mixed models due to the high proportion of samples with no detectable levels of IgG at baseline. Without this correction, it would not be possible to assume that the IgG levels data were normally distributed. This is a two-part model, one part is the conditional model that fits a Gaussian mixed model for the non-zero observations (detectable levels of IgG antibody), and a zero-inflated model that fits the binary model for the probability of observing a zero or not (detectable vs non-detectable IgG antibody levels).

We fitted the log10-transformed IgG levels in the zero-inflated Gaussian mixed model with subject level random effects for both intercept and slope across days from vaccination. The days from vaccination were represented with four natural cubic splines (NCS) to allow the modeling of non-linear trends. The knots of the four NCS were defined based on the 25%, 50%, and 75% quantiles of the days from vaccination. We fitted the model that included the four NCS, N protein seropositivity, and BMI groups. In addition, we included a two-way interaction between NCS and N protein seropositivity and NCS and BMI groups. For the zero-inflated model, we included days from vaccination and N protein seropositivity as the independent variables.

2.6. IgG antibody levels associated with the interval of days between BNT162b2 vaccine doses: Zero-inflated Gaussian mixed model

We utilized zero-inflated Gaussian mixed models due to the high proportion of samples with no detectable levels of IgG at baseline. Without this correction, it would not be possible to assume that the IgG levels data were normally distributed. This is a two-part model, one part is the conditional model that fits a Gaussian mixed model for the non-zero observations (detectable levels of IgG antibody), and a zero-inflated model that fits the binary model for the probability of observing a zero or not (detectable vs non-detectable IgG antibody levels).

We fitted the log10-transformed IgG levels in the zero-inflated Gaussian mixed model with subject level random effects for both intercept and slope across days from vaccination. The days from vaccination were represented with four NCS to allow the modeling of non-linear trends. The knots of the four NCS were defined based on the 25%, 50%, and 75% quantiles of the days from vaccination. We fitted the model that included the four NCS, N protein seropositivity, the dosing interval, and age groups. In addition, we included a two-way interaction between NCS and N protein seropositivity, NCS and dosing interval, NCS and age groups, N protein seropositivity and dosing interval, and N protein seropositivity and age groups. For the

zero-inflated model, we included days from vaccination and N protein seropositivity as the independent variables.

2.7. IgA antibody responses associated with the interval of days between BNT162b2 vaccine doses: Binomial generalized mixed model

We utilized binomial generalized mixed models to model the IgA responses over time after vaccination. We fitted binary responses of positive IgA response with subject level random effects for intercept. The days from vaccination were represented with four NCS to allow the modeling of non-linear trends. The knots of the four NCS were defined based on the 25%, 50%, and 75% quantiles of the days from vaccination. We fitted the model that included the four NCS, N protein seropositivity, and the dosing interval. In addition, we included a two-way interaction between NCS and N protein seropositivity, NCS and dosing interval, and N protein seropositivity and dosing interval.

2.8. IgG antibody levels associated with IFN-γ levels: Zero-inflated Gaussian mixed model

We utilized zero-inflated Gaussian mixed models due to the high proportion of samples with no detectable levels of IgG at baseline. Without this correction, it would not be possible to assume that the IgG levels data were normally distributed. This is a two-part model, one part is the conditional model that fits a Gaussian mixed model for the non-zero observations (detectable levels of IgG antibody), and a zero-inflated model that fits the binary model for the probability of observing a zero or not (detectable vs non-detectable IgG antibody levels).

We fitted the log10-transformed IgG levels in the zero-inflated Gaussian mixed model with subject level random effects for both intercept and slope across days from vaccination. The days from vaccination were represented with four NCS to allow the modeling of non-linear trends. The knots of the four NCS were defined based on the 25%, 50%, and 75% quantiles of the days from vaccination. We fitted the model that included the four NCS, N protein seropositivity, vaccine administrated, and IFN-γ levels. In addition, we included a two-way interaction between NCS and N protein seropositivity, NCS and IFN-γ levels, and N protein seropositivity and IFN-γ levels. For the zero-inflated model, we included days from vaccination and N protein seropositivity as the independent variables.

Supplementary Figures

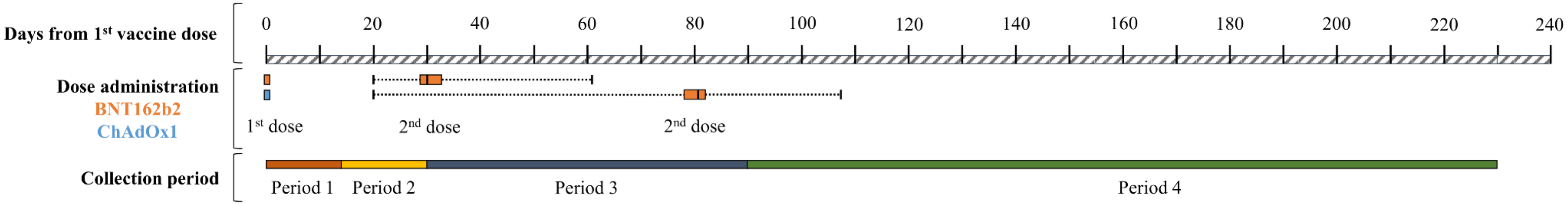

Supplementary Figure 1. Schematic illustration of sample collection timeline and the administration of the vaccine doses. Blood samples were collected at baseline (period 1), approximately 3 weeks after the first vaccine dose (period 2), approximately 2 months after the first vaccine dose (period 3), and approximately 6 months after the first vaccine dose (period 4). At time 0 it was administrated either the first dose of BNT162b2 (orange) or the first dose of ChAdOx1 (blue). Individuals vaccinated with the first dose of BNT162b2 received the second dose of BNT162b2 after a median of 30 days (IQR: 20–33 days). Individuals vaccinated with the first dose of ChAdOx1 received the second dose of BNT162b2 after a median of 81 days (IQR: 80–83 days. Dotted lines indicate the minimum and maximum range of days after the first dose. IQR: interquartile range.

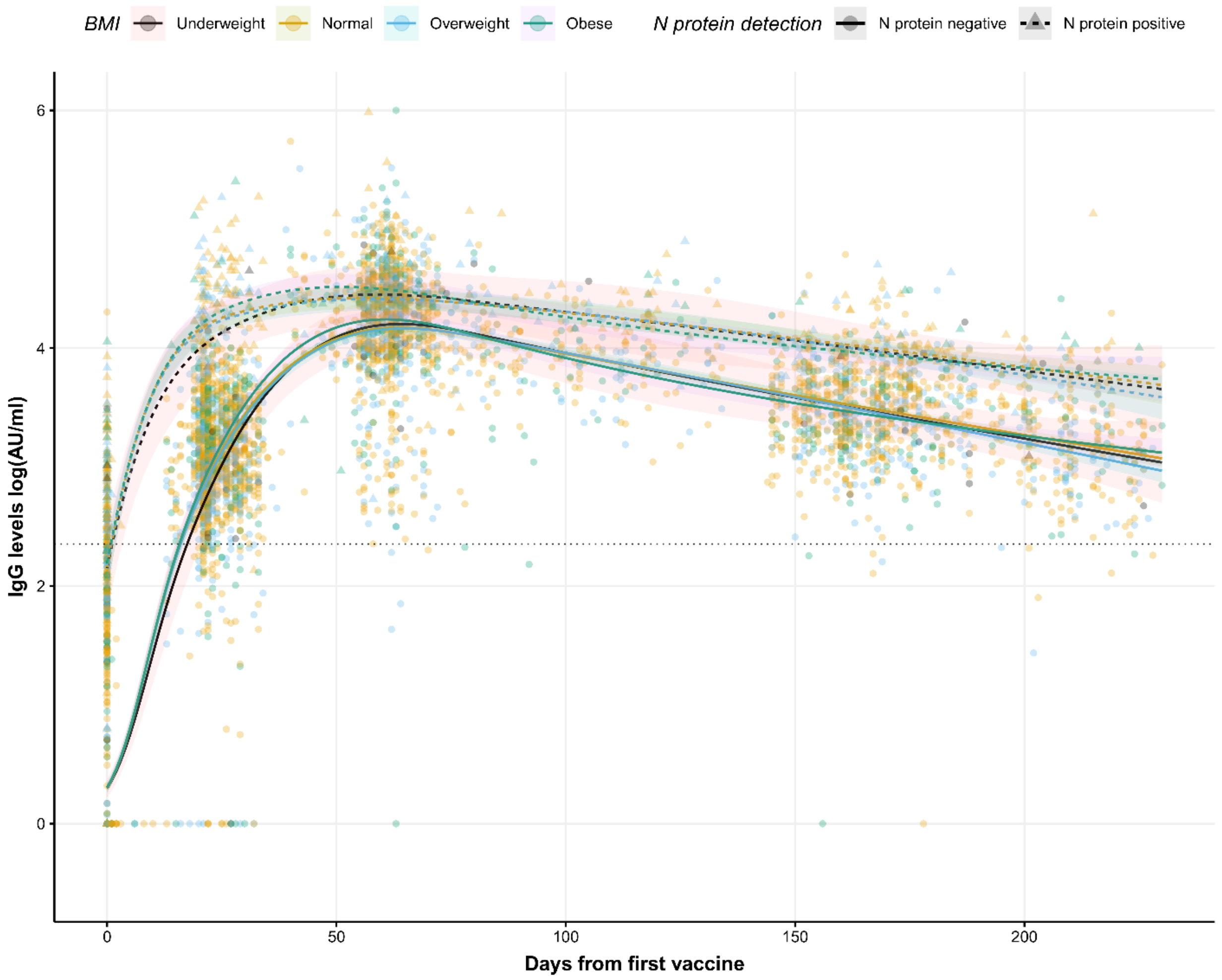

Supplementary Figure 2. Correlation of the BMI and circulating IgG levels against RBD after the first dose of the COVID-19 vaccination. Distribution of IgG levels, represented in log(AU/ml), over time (days from the first vaccine) in individuals vaccinated against COVID-19. Circles and triangles represent the observed levels of circulating IgG antibodies for non-previously infected and infected individuals with SARS-CoV-2, respectively. Solid and dashed lines represent the predicted levels of circulating IgG antibodies for non-previously infected and infected individuals with SARS-CoV-2, respectively. Black, yellow, light blue, and dark blue colors represent underweight, normal, overweight, and obese individuals, respectively. Black dotted line represents the threshold for assay positivity. Shadowed areas represent the 95% confidence interval. Centre for the confidence interval is the predicted (mean) values.

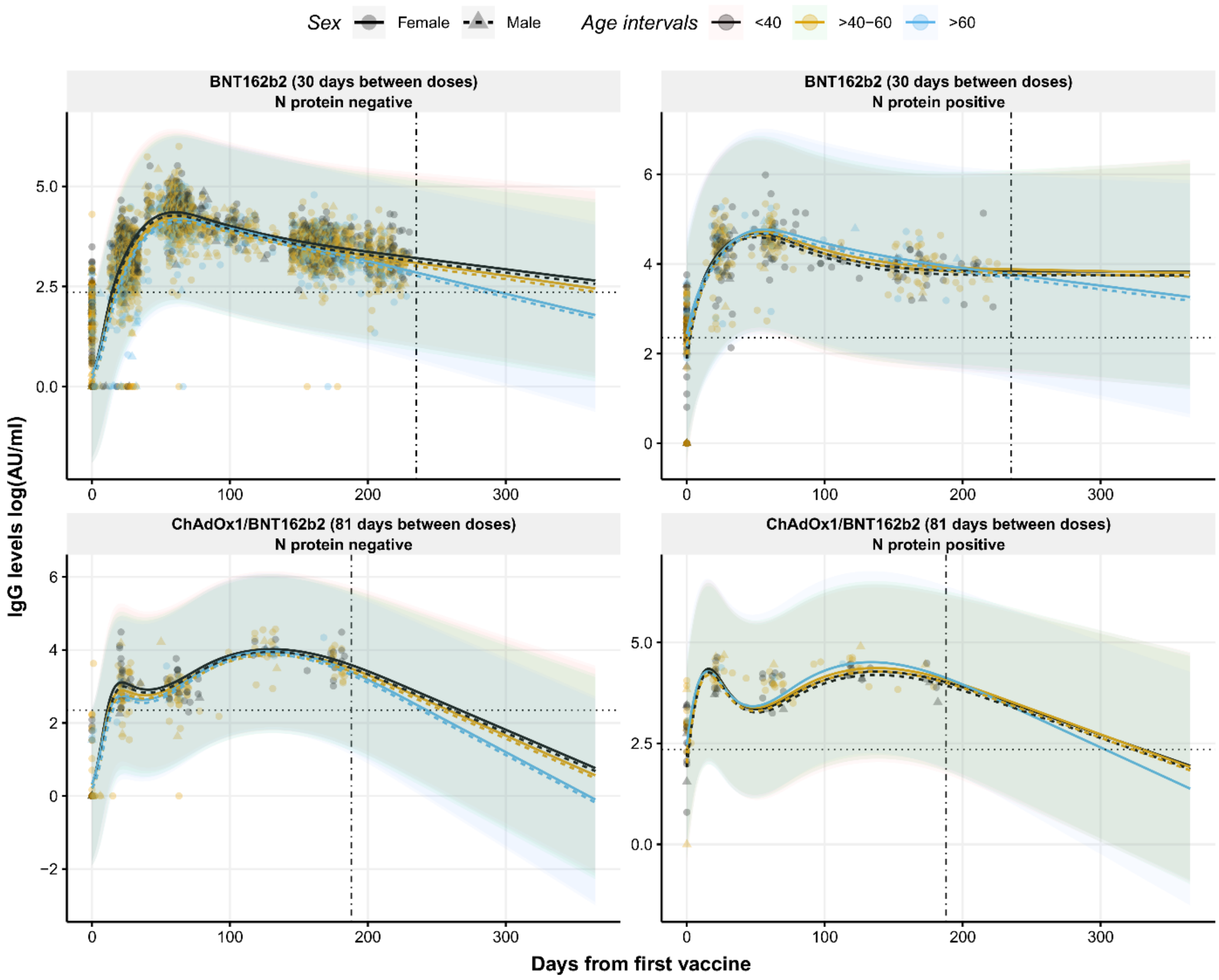

Supplementary Figure 3. Dynamics and projection with prediction intervals of circulating IgG levels against RBD after the first dose of the COVID-19 vaccination using a non-linear model. Distribution of IgG levels, represented in log(AU/ml), over time (days from the first vaccine) in individuals with no prior infection vaccinated with BNT162b2 (top left) or with the combination ChAdOx1/BNT162b2 (bottom left), and in individuals previously infected and vaccinated with BNT162b2 (top right) or with the combination ChAdOx1/BNT162b2 (bottom right). Circles and triangles represent the observed levels of circulating IgG antibodies in females and males, respectively. Solid and dashed lines represent the predicted levels of circulating IgG antibodies calculated by the model in females and males, respectively. Black, yellow, and blue colors represent individuals with age <40, 40–60, and >60 years, respectively. Horizontal black dotted line represents the threshold for assay positivity. Vertical dash-dotted line indicates where the out-of-sample trend starts. Shadowed areas represent the 95% prediction interval. Centre for the confidence interval is the predicted (mean) values.

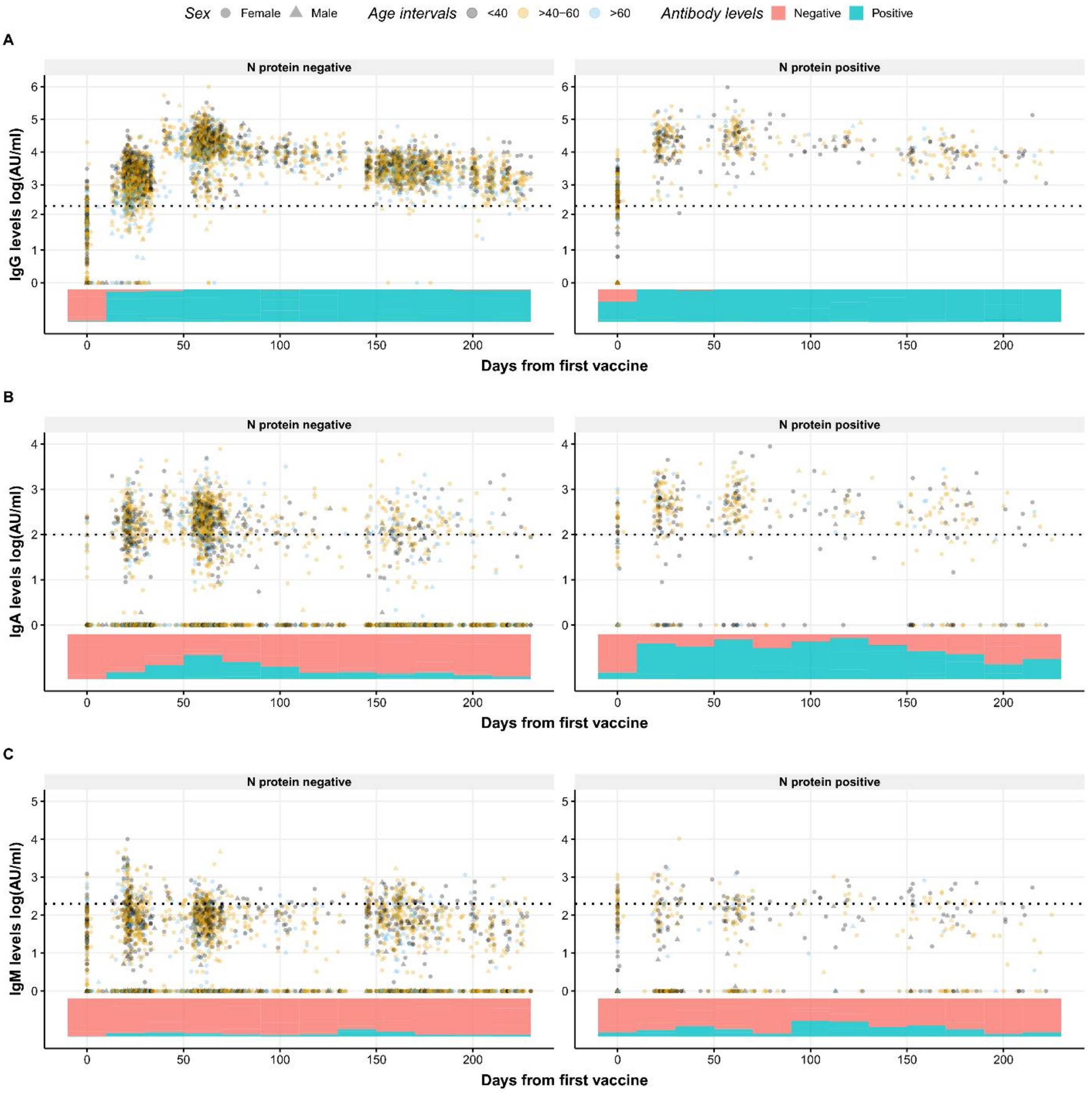

Supplementary Figure 4. Observed levels and positive responses of circulating IgG, IgM, and IgA against RBD after the first dose of the COVID-19 vaccination. Distribution of IgG levels, represented in log (AU/ml), over time (days from the first vaccine) in individuals with no prior infection (left) or previously infected (right). Bottom histogram represents the relative frequency of positive IgG responses over time (**A**). Distribution of IgA levels, represented in log (AU/ml), over time (days from the first vaccine) in individuals with no prior infection (left) or previously infected (right). Bottom histogram represents the relative frequency of positive IgA responses over time (**B**). Distribution of IgM levels, represented in log (AU/ml), over time (days from the first vaccine) in individuals with no prior infection (left) or previously infected (right). Bottom histogram represents the relative frequency of positive IgM responses over time (**C**). Circles and triangles represent the observed levels of circulating antibodies in females and males, respectively. Black, yellow, and blue colors represent individuals with age <40, 40–60, and >60 years, respectively. Horizontal black dotted line represents the threshold for assay positivity. Blue and pink backgrounds represent the conditional density estimation of positive and negative antibody responses, respectively.

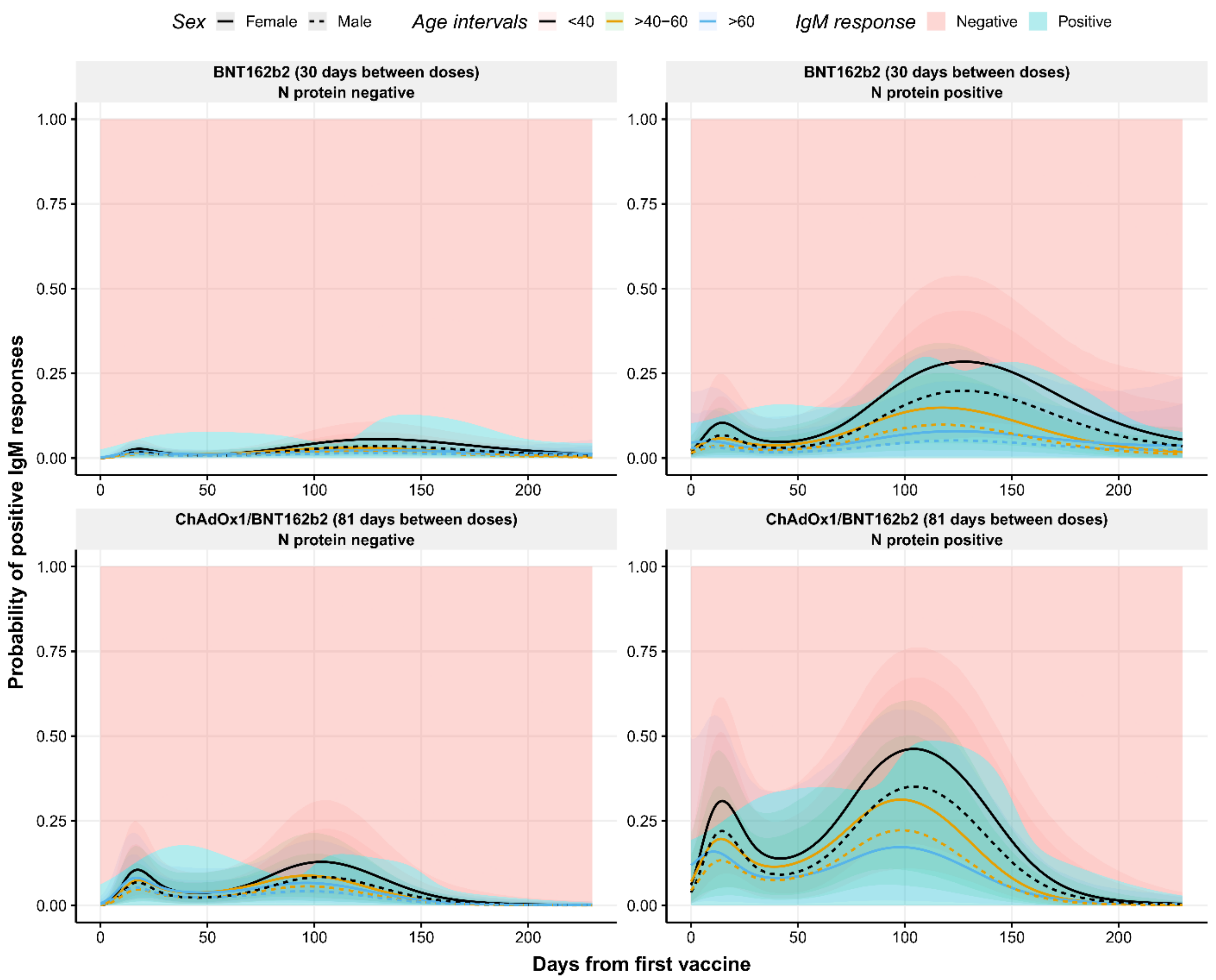

Supplementary Figure 5. Observed and predicted probability of positive IgM responses against RBD after the first dose of the COVID-19 vaccination. Distribution of positive IgM response (probability) over time (days from the first vaccine) in individuals with no prior infection vaccinated with BNT162b2 (top left) or with the combination ChAdOx1/BNT162b2 (bottom left), and in individuals previously infected and vaccinated with BNT162b2 (top right) or with the combination ChAdOx1/BNT162b2 (bottom right). Blue and pink backgrounds represent the conditional density estimation of positive and negative IgM responses, respectively. Solid and dashed lines represent the predicted probability of positive IgM responses calculated by the model in females and males, respectively. Black, yellow, and blue colors represent individuals with age <40, 40–60, and >60 years, respectively. Shadowed areas represent the 95% confidence interval. Centre for the confidence interval is the predicted (mean) values.

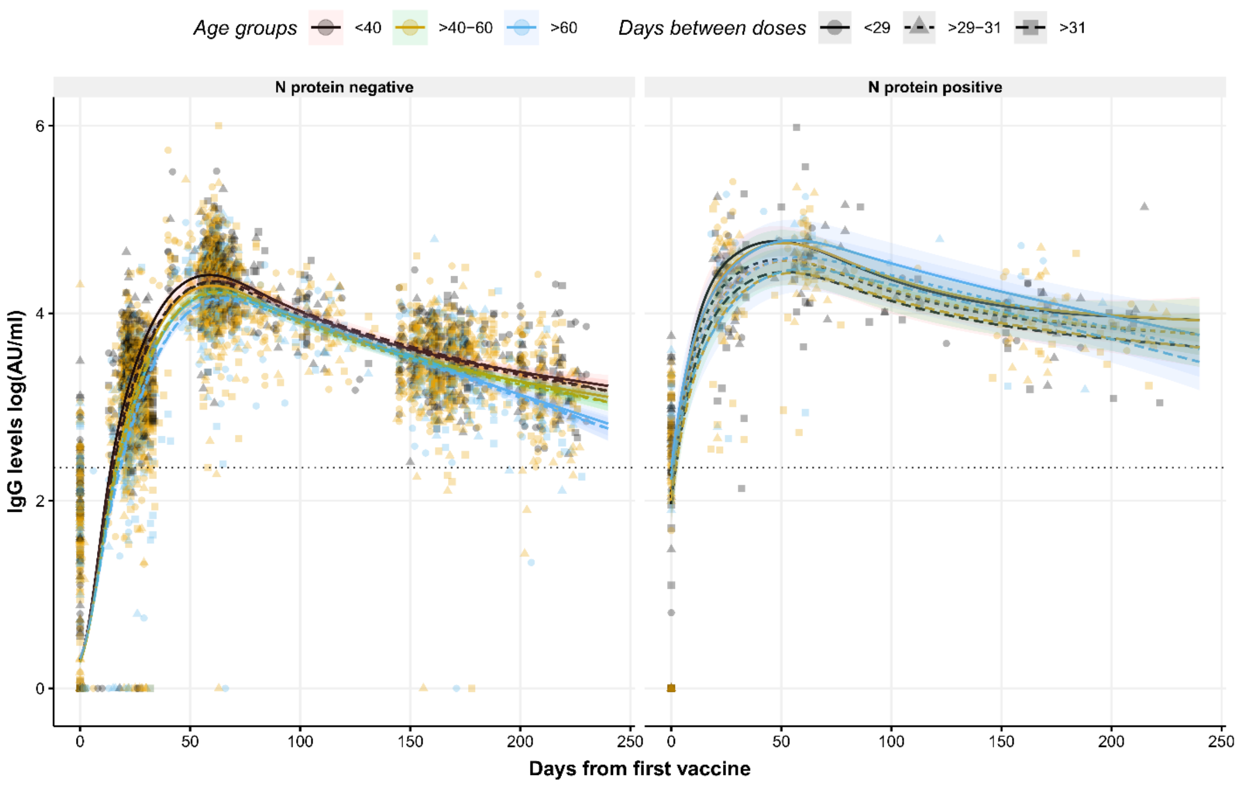

Supplementary Figure 6. Effect of the time of administration of the second dose of the BNT162b2 vaccine in the dynamic of circulating IgG levels against RBD. Distribution of IgG levels, represented in log(AU/ml), over time (days from the first vaccine) in individuals vaccinated with BNT162b2 with no prior infection (left) and previously infected with SARS-CoV-2 (right). Circles, triangles, and squares represent the observed levels of circulating IgG antibodies for individuals with an interval between doses of <29, 29–31, and >31 days, respectively. Solid, short dashed, and long dashed lines represent the predicted levels of circulating IgG levels for individuals with an interval between doses of <29, >29–31, and >31 days, respectively. Black, yellow, and blue colors represent individuals with age <40, >40–60, and >60 years, respectively. Black dotted line represents the threshold for assay positivity. Shadowed areas represent the 95% confidence interval. Centre for the confidence interval is the predicted (mean) values.

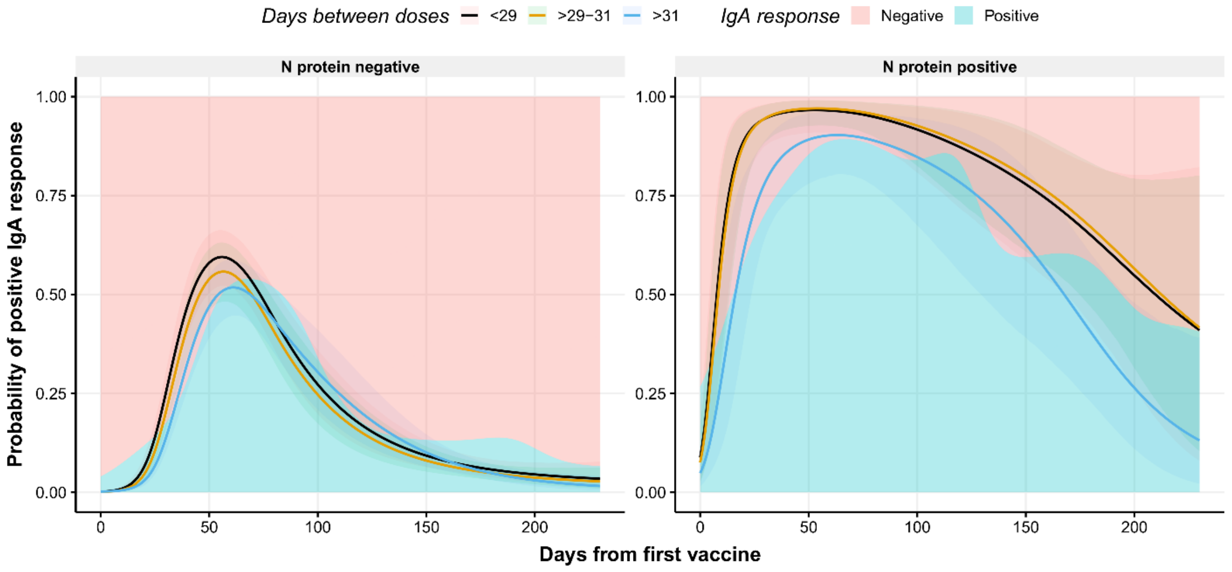

Supplementary Figure 7. Effect of the time of administration of the second dose of the BNT162b2 vaccine in positive IgA responses against RBD. Distribution of positive IgA responses (probability) over time (days from the first dose) in individuals vaccinated with BNT162b2 non-previously infected (left) or previously infected (right). Blue and pink backgrounds represent the conditional density estimation of positive and negative IgA responses, respectively. Solid lines represent the predicted levels of positive IgA responses over time. Black, yellow, and blue colors represent individuals with an interval between doses of <29, 29–31, and >31 days, respectively. Shadowed areas represent the 95% confidence interval. Centre for the confidence interval is the predicted (mean) values.

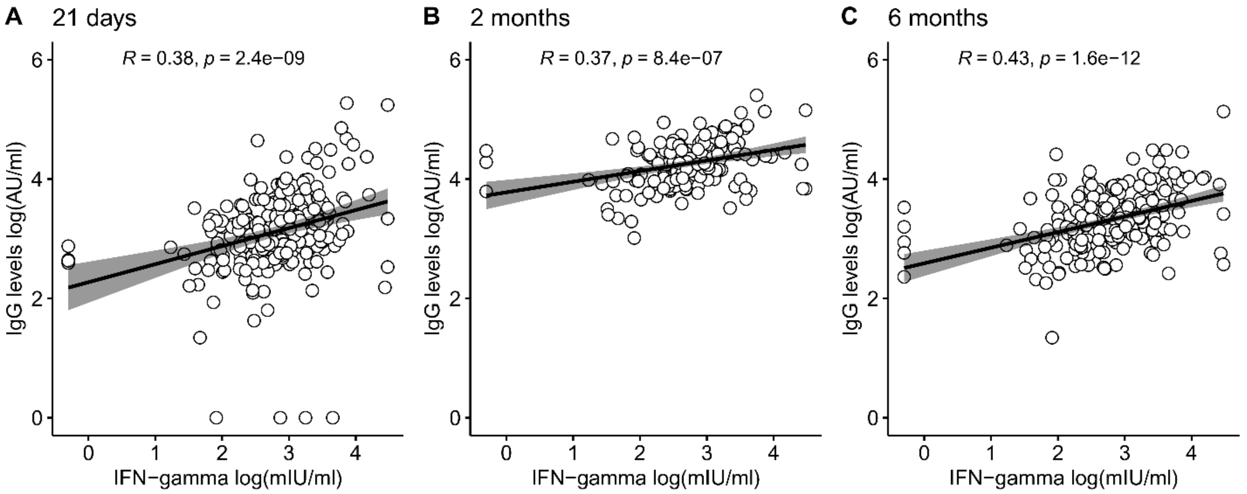

Supplementary Figure 8. Correlation between released IFN-γ from activated T-cells upon recognition of peptides derived from the S1 subunit of S protein and IgG levels. Correlation between IFN-γ collected at approximately 6 months after the first dose with IgG levels measured at (**A**) approximately 21 days, n=237 individuals; (**B**) approximately 2 months, n=164 individuals; and (**C**) approximately 6 months approximately, n=250 individuals. Spearman rank correlation was performed (two-sided). P<0.05 was considered significant. Shadowed areas represent the 95% confidence interval.

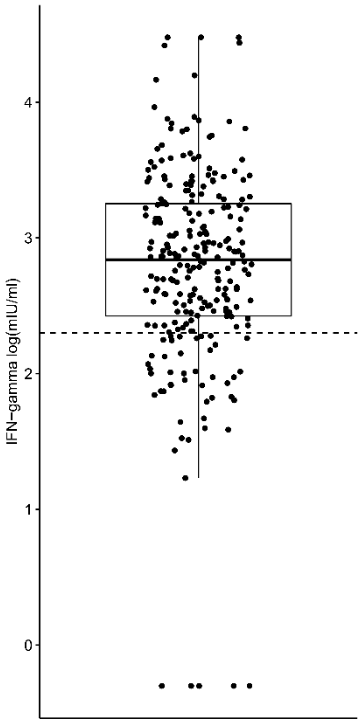

Supplementary Figure 9. Distribution of IFN-γ levels from activated T-cells upon recognition of peptides derived from the S1 subunit of S protein. IFN-γ levels collected approximately after 6 months from vaccination are represented in log(mIU/ml). Data reported as the median and interquartile range (box), whiskers represent 1.5 times the interquartile range. Dashed horizontal line indicates the threshold for assay positivity. Dots represent the observed data from 250 participants.

Supplementary Tables

Supplementary Table 1. RT-PCR responses in natural infection primed individuals

	**N protein positive (N = 161)**
RT-PCR response, received	105 (65.2%)
Before first dose	91 (56.5%)
Between first and second dose	14 (8.7%)
RT-PCR response, not analyzed	56 (34.8%)

Supplementary Table 2. Multiple linear regression analysis for IFN-γ levels associations with sex, previous infection, age, IgG levels, and IgA responses in vaccinated individuals

**Variables**	**Estimate**	**Std. Error**	***p*-value**
Intercept[a]	1.45601	0.34004	2.67e-05 ***
Sex, male	-0.23172	0.14394	0.10873
N protein serology, positive	0.51852	0.17200	0.00285 **
Age, >40–60 years old	-0.19829	0.09436	0.03663 *
Age, >60 years old	-0.30405	0.14198	0.03322 *
IgG levels, logAU/ml	0.43605	0.09892	1.57e-05 ***
IgA response, positive	-0.01965	0.17320	0.90978

[a]Intercept defined as a <40 years old female individual, non-previously infected.
* p < 0.05, ** p < 0.01, **** p < 0.0001 (two-sided p-values were calculated)

Supplementary References

1. Brooks M, Kirstensen K, Van Bethem KJ et al. glmmTMB balances speed and flexibility among packages for zero-inflated generalized linear mixed modeling. The R Journal 2017;9(2):378-400.
