# Supplementary material for: Modeling of waning immunity after SARS-CoV-2 vaccination and influencing factors
Source: Nat Commun. 2022 Mar 28;13:1614. doi: 10.1038/s41467-022-29225-4 (PMC8960902; doi:10.1038/s41467-022-29225-4)
Supplement: Supplementary file 3 — Reporting Summary [file 41467_2022_29225_MOESM3_ESM.pdf]

nature portfolio

Corresponding author(s): Peter Garred
Last updated by author(s): 23.02.2022

Reporting Summary

Nature Portfolio wishes to improve the reproducibility of the work that we publish. This form provides structure for consistency and transparency in reporting. For further information on Nature Portfolio policies, see our Editorial Policies and the Editorial Policy Checklist.

Statistics

For all statistical analyses, confirm that the following items are present in the figure legend, table legend, main text, or Methods section.

n/a	Confirmed	
☐	☒	The exact sample size (*n*) for each experimental group/condition, given as a discrete number and unit of measurement
☐	☒	A statement on whether measurements were taken from distinct samples or whether the same sample was measured repeatedly
☐	☒	The statistical test(s) used AND whether they are one- or two-sided<br>*Only common tests should be described solely by name; describe more complex techniques in the Methods section.*
☐	☒	A description of all covariates tested
☐	☒	A description of any assumptions or corrections, such as tests of normality and adjustment for multiple comparisons
☐	☒	A full description of the statistical parameters including central tendency (e.g. means) or other basic estimates (e.g. regression coefficient) AND variation (e.g. standard deviation) or associated estimates of uncertainty (e.g. confidence intervals)
☐	☒	For null hypothesis testing, the test statistic (e.g. *F*, *t*, *r*) with confidence intervals, effect sizes, degrees of freedom and *P* value noted<br>*Give P values as exact values whenever suitable.*
☒	☐	For Bayesian analysis, information on the choice of priors and Markov chain Monte Carlo settings
☒	☐	For hierarchical and complex designs, identification of the appropriate level for tests and full reporting of outcomes
☒	☐	Estimates of effect sizes (e.g. Cohen's *d*, Pearson's *r*), indicating how they were calculated

Our web collection on statistics for biologists contains articles on many of the points above.

Software and code

Policy information about availability of computer code

Data collection	R (version 4.1.0 for Windows, R Foundation for Statistical, Computing). Packages used: readxl (version 1.3.1), dplyr (version 1.0.7), tidyr (version 1.1.4), lubridate (version 1.8.0), reshape (version 0.8.8), table1 (version 1.4.2), ggpubr (version 0.4.0), effects (version 4.2.0), wesanderson (version 0.3.6), ggthemes (version 4.2.4), ggnewscale (version 0.4.5), gridExtra (version 2.3), cowplot (version 1.1.1), forcats (version 0.5.1).<br>GraphPad Prism version 9.2.0 (GraphPad Software, La Jolla, CA).
Data analysis	R (version 4.1.0 for Windows, R Foundation for Statistical, Computing). Packages used: glmmTMB (version 1.1.2.3), DHARMa (version 0.4.4), lmerTest (version 3.1.3), splines (version 4.0.3), car (version 3.0.11), lme4 (version 1.1.27.1).<br><br>Code used in this study is deposited in Zenodo database: https://doi.org/10.5281/zenodo.6234433

For manuscripts utilizing custom algorithms or software that are central to the research but not yet described in published literature, software must be made available to editors and reviewers. We strongly encourage code deposition in a community repository (e.g. GitHub). See the Nature Portfolio guidelines for submitting code & software for further information.

Data

Policy information about availability of data

All manuscripts must include a data availability statement. This statement should provide the following information, where applicable:

- Accession codes, unique identifiers, or web links for publicly available datasets
- A description of any restrictions on data availability
- For clinical datasets or third party data, please ensure that the statement adheres to our policy

The data used in this study are available in the Zenodo database [https://doi.org/10.5281/zenodo.6234161].

Field-specific reporting

Please select the one below that is the best fit for your research. If you are not sure, read the appropriate sections before making your selection.

☒ Life sciences ☐ Behavioural & social sciences ☐ Ecological, evolutionary & environmental sciences

For a reference copy of the document with all sections, see nature.com/documents/nr-reporting-summary-flat.pdf

Life sciences study design

All studies must disclose on these points even when the disclosure is negative.

Sample size	The sample size was defined by including in the analyses all the individuals who had administered two vaccine doses and had at least three or more visits, independently of previous natural infection.
Data exclusions	Individuals who had a natural infection and the baseline sample was collected more than 14 days before the administration of the first vaccine dose had the baseline time point excluded from analysis.<br>Individuals with two or less visits were excluded from the analysis.<br>Individuals with partial vaccination were excluded from the analysis.<br>Individuals partially vaccinated with Spikevax (Moderna) or fully vaccinated with ChAdOx1 vaccine (AstraZeneca/Oxford) were excluded from the analysis due to the low sample number (n=8 and n=1, respectively).
Replication	Each sample has been analyzed once. Analytical reproducibility is demonstrated by the analysis of serial samples collected from the same individuals included in the study (minimum 3 serial samples collected per individual).<br>The R code used for the statistical analysis is publicly accessible ensuring statistical reproducibility.
Randomization	All samples delivered for experimental analysis were performed in a randomized manner.
Blinding	All assays as well as the analytical analysis have been performed blinded.

Reporting for specific materials, systems and methods

We require information from authors about some types of materials, experimental systems and methods used in many studies. Here, indicate whether each material, system or method listed is relevant to your study. If you are not sure if a list item applies to your research, read the appropriate section before selecting a response.

Materials & experimental systems

n/a	Involved in the study	
☐	☒	Antibodies
☒	☐	Eukaryotic cell lines
☒	☐	Palaeontology and archaeology
☒	☐	Animals and other organisms
☐	☒	Human research participants
☒	☐	Clinical data
☒	☐	Dual use research of concern

Methods

n/a	Involved in the study	
☒	☐	ChIP-seq
☒	☐	Flow cytometry
☒	☐	MRI-based neuroimaging

Antibodies

Antibodies used	HRP-conjugated polyclonal rabbit anti-human IgG. Supplier: Agilent Technologies. Catalogue number: P0214. Batch: 20079450.<br>HRP-conjugated polyclonal rabbit anti-human IgM. Supplier: Agilent Technologies. Catalogue number: P0215. Batch: 20073908.<br>HRP-conjugated polyclonal rabbit anti-human IgA. Supplier: Agilent Technologies. Catalogue number: P0216. Batch: 20073902.<br>SARS-CoV-2 Spike S1 Antibody, Human Chimeric IgG. Supplier: Genscript. Clone: HC2001. Catalogue number: A02038-100. Batch: 20E002152.

	SARS-CoV-2 Spike S1 Antibody, Human Chimeric IgM. Supplier: Genscript. Clone: hIgM2001. Catalogue number: A02046-100. Batch: 20E002153.<br>SARS-CoV-2 Spike S1 Antibody, Human Chimeric IgA. Supplier: Genscript. Clone: hIgA2001. Catalogue number: A02071-100. Batch: B2010012.<br>Antibodies used for IFN-γ quantification were included in the commercial Quan-T-Cell ELISA kit. Supplier: EUROIMMUN. Catalogue number: EQ 6841-9601. Batches: E210315CB and E210604AQ.
Validation	HRP-conjugated polyclonal rabbit anti-human IgG, IgM and IgA validation details are described in the manufacturer's user manual:<br><br>- IgG: https://www.agilent.com/cs/library/packageinsert/public/SSP0214CEEFG_01.pdf;<br>- IgM: https://www.agilent.com/cs/library/packageinsert/public/SSP0215CEEFG_01.pdf;<br>- IgA: https://www.agilent.com/cs/library/packageinsert/public/SSP0216CEEFG_01.pdf).<br><br>As well, we have previously validated these antibodies in our assay on a previous publication: The Journal of Immunology, DOI: https://doi.org/10.4049/jimmunol.2000898).<br><br>SARS-CoV-2 Spike S1 Antibody, Human Chimeric IgG, IgM and IgA validation details are described in the manufacturer's user manual:<br><br>- IgG: https://www.genscript.com/product/documents?cat_no=A02038&catalogtype=Document-PROTOCOL;<br>- IgM: https://www.genscript.com/product/documents?cat_no=A02046&catalogtype=Document-PROTOCOL;<br>- IgA: https://www.genscript.com/product/documents?cat_no=A02071&catalogtype=Document-PROTOCOL).<br><br>The Quant-T-Cell ELISA kit validation details are described in the manufacturer's data sheet:<br><br>https://www.coronavirus-diagnostics.com/documents/Indications/Infections/Coronavirus/ET_2606_D_UK_A.pdf)

Human research participants

Policy information about studies involving human research participants

Population characteristics	The population analyzed (n = 1754) is comprised of health care professionals from Rigshospitalet and Herlev-Gentofte University Hospital (Capital Region of Denmark). The 86.4% of the population is female at a median age of 49 (IQR: 39–58) years and a median BMI of 24 (IQR: 22–27). A total of 161 individuals had a previous SARS-CoV-2 infection.
Recruitment	Participants were recruited via email and e-Boks, a trusted Nordic platform of digital postboxes. Invitations were sent to every health care individual employeed at Rigshospitalet and Herlev-Gentofte University Hospital (Capital Region of Denmark) and participants freely volunteered to participate in the study. Volunteers older than 18 years old without a diagnosed pathology was included in the study. Sample collection did not interfere with the vaccination strategy.
Ethics oversight	The Regional Scientific Ethics Committee of the Capital Region of Denmark approved the study (H-20079890)

Note that full information on the approval of the study protocol must also be provided in the manuscript.
